# The Impact of Climate, Sulfur Dioxide, and Industrial Dust on δ^18^O and δ^13^C in Glucose from Pine Tree Rings Growing in an Industrialized Area in the Southern Part of Poland

**DOI:** 10.1007/s11270-016-2808-0

**Published:** 2016-03-12

**Authors:** Barbara M. Sensuła

**Affiliations:** Institute of Physics-Center for Science and Education, Silesian University of Technology, Konarskiego 22B, 44-100 Gliwice, Poland

**Keywords:** Pine, Stable oxygen isotopes, Stable carbon isotopes, Bio-indicators, Dust, Sulfur dioxide, Climate

## Abstract

The mass spectrometric analysis of the impact of sulfur dioxide and dust emission on carbon and oxygen stable isotopic compositions of glucose hydrolysed from α-cellulose samples extracted from Scots pine growing in the vicinity of “Huta Katowice” steelworks was the main aim of this study. The annual rings covered the time span from 1975 to 2012 AD. The relationships between climatic conditions, sulfur dioxide, and industrial dust emission and oxygen and carbon isotopic compositions were analyzed using correlation function methods. This study shows the first analysis of carbon and oxygen stable isotopes in glucose as the bio-indicators of CO_2_, sulfur dioxide, and industrial dust emission. The anticoincidence trend of δ^18^O and δ^13^C and dust and sulfur dioxide confirms that the decreases of dust and sulfur dioxide industrial emission increase δ^18^O and δ^13^C values in glucose.

## Introduction

The pollutant emission affects the health of the population (Absalon and Ślesak 2010) and quality of life (Absalon and Ślesak 2012). The exposure industrial dust emission can cause inter alia asthma bronchitis, lung damage, cancer, heavy metal poisoning, and cardiovascular effects including heart attack and premature death in human life. Sulfur dioxide emission can cause the eye irritation, wheezing, chest tightness, shortness of breath, and lung damage. The emission of pollution also has an impact on the environment and the condition of the forests (Breymeyer 1998). It can cause the following changes in the environment: visibility impairment, plant and water damage, and aesthetic damage. Sulfur dioxide emission contributes to the formation of acid rain. The southern part of Poland is one of the regions with the highest levels of air pollution in Europe, where the highest levels of dust and gaseous pollutants were recorded in the late 1970s (Marland et al. 2008). A reduction of industrial pollution emissions in Poland, similarly as in eastern Europe, is connected with the modernization of the industrial sector and also with EU legislation and the application of restrictive governmental regulations on emissions. In Poland, the monitoring of air pollution is generally limited to the last decades.

Annual tree rings of Scots pine (*Pinus sylvestris* L.) are useful in bio-monitoring of environment with annual or sub-annual resolution. Isotopic fractionation occurs during physiological processes responsible for plant growth (e.g., Craig and Gordon 1965; Dongmann et al. 1974; Farquhar and Lloyd 1993; Saurer et al. 2002; Barbour et al. 2000, 2002; McCarroll and Loader 2004; Sensuła 2015), and the isotopic composition of pine tree rings can be used to study the climatic changes and the impact of human activity on ecosystem in the past to better understand future consequences of ecosystem changes (Schweingruber 1996; De Vries et al. 2000; McCarroll and Loader 2004; McCarroll et al. 2009; Pazdur et al. 2007, 2013; Sensuła et al. 2011a, b; Sensuła and Pazdur 2013a, b; Sensuła et al. 2015a, b; Sensuła 2015). During tree growth, gaseous compounds (also air pollutants) are assimilated via leaves. Garsed et al. (1979) reported that the conifers may be the most sensitive plants to SO_2_. Through photosynthesis, plants convert CO_2_ and H_2_O to glucose (C_6_H_12_O_6_), using light, and release oxygen to the atmosphere. Although the carbon of each annual tree ring has its origin in the CO_2_ of air, and oxygen come from soil water and thus precipitation, the isotopic ratios in wood are very different to those in either air or water, so trees do not passively collect and store these elements. The primary value of the isotopic records in tree rings is not simply as samples of ancient air or water but as sensitive bio-indicators of the way that the components of air and water have been changed by the trees in response to the environments in which they have grown (McCarroll and Loader 2004).

The carbon isotopic composition (δ^13^C) of trees is influenced by carbon isotopic composition (δ^13^C) of atmospheric CO_2_, diffusion of CO_2_ through stomata, and enzymatic discrimination during the irreversible step of CO_2_ fixation (Roden and Ehleringer 1999; Roden et al. 2000; Roden 2008). The carbon isotopic composition in plant varies with water stress and solar radiation and has been correlated inter alia with rainfall amount, vapor pressure deficit, canopy position, and hydraulic conductivity associated with tree height (Ehleringer 1990; Ehrelinger and Vogel 1993; Comstock and Ehleringer 1992; Buchmann et al 2002; Yoder et al. 1994).

The oxygen isotopic composition of trees is influenced inter alia by the δ^18^O in meteoric water, the δ^18^O of atmospheric vapor, atmospheric humidity, and vapor pressure deficit (Farquhar and Lloyd 1993; Roden et al. 2000). The δ^18^O values in plant have been correlated with air temperature, relative humidity, transpiration rates, and water balance, presumably all through contributions of source water (the xylem water in suberized stems) and leaf water enrichment (Burk and Stuiver 1981; Edwards and Fritz 1986; White et al. 1994; Barbour et al. 2000).

The isotopic compositions of plants are influenced by emission of atmospheric pollution (Savard 2010), such as, for example, sulfur dioxide (Dongmann et al. 1974; Ehleringer 1990; Flanagan et al. 1991; Hill et al. 1995; Roden and Ehleringer 1999; Roden et al. 2000; Roden 2008; Barbour et al. 2000, 2002; Rinne et al 2010) and carbon dioxide (Craig 1954; McCarroll and Loader 2004; McCarroll et al. 2009; Pazdur et al. 2007, 2013; Sensuła et al. 2011a, b; Sensuła and Pazdur 2013a, b; Sensuła 2015).

Previous studies have shown weakened climate signal in δ^13^C and δ^18^O of α-cellulose of tree ring cellulose as well as deviant trends between δ^13^C and δ^18^O series caused by SO_2_ pollution during the twentieth century (e.g., Rinne et al. 2010; Boettger et al. 2014). Since the 1970s, most ecological and environmental studies have concentrated on the analysis of α-cellulose, a linear homopolymer built from β-1,4-linked glucose units, as the dominant and most easily isolated wood component (Craig 1954; Libby and Pandolfi 1974; Leavitt and Long 1982, 1988; Ehrelinger and Vogel 1993; McCarroll and Loader 2004; McCarroll et al. 2009; Pazdur et al. 2007, 2013; Sensuła et al. 2011a, b; Sensuła and Pazdur 2013a, b). In terms of the literature background, there are few studies concerning carbon and oxygen isotopic fractionation in glucose (e.g., Sensuła et al. 2009, 2011a, b; Sensuła and Pazdur 2013a).

Sulfur dioxide and industrial dust emissions may also potentially influence carbon and oxygen stable isotope compositions in glucose. To compare the nature of the air pollution signal and its influence on isotopic fractionation in glucose of pine trees, two forests were selected as a study area: (i) a stand where the influence of sulfur dioxide and dust industrial emission to the atmosphere is significant and (ii) a natural forest located ca. 100 km from industrial source of pollution.

The main aim of this study is the mass spectrometric investigation of the impact of climate, sulfur dioxide, and dust emission on the carbon and oxygen isotopic compositions of glucose hydrolysed from α-cellulose samples extracted from Scots pine (*P. sylvestris* L.) growing in the vicinity of “Huta Katowice” steelworks (Silesia, Poland). This kind of investigation with using trees as bio-indicators is still lacking in Poland and in east and central Europe. More studies are needed to better understand the impact of atmospheric pollution in the past and present on European tree ring isotope chronologies. This study is a part of a large-scale research project (BIOPOL).

## Materials and Method

### Sampling Sites

**Fig. 1:**
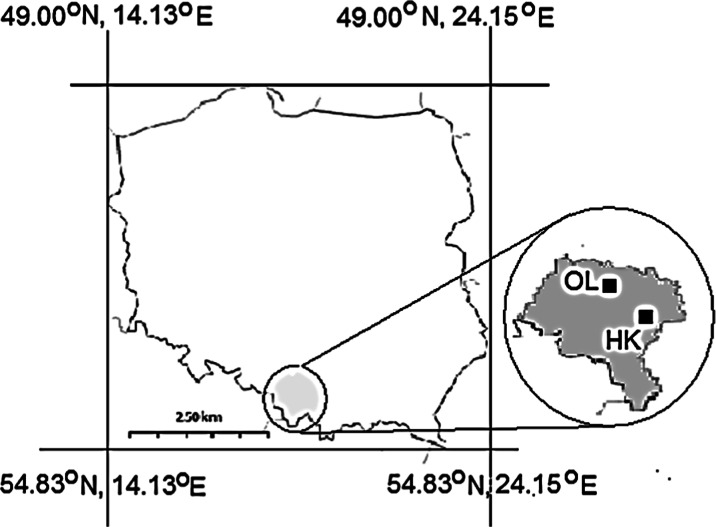
Sampling sites in the south part of Poland: polluted site nearby “Huta Katowice” (HK) and comparative site in Olesno (OL)

**Fig. 2:**
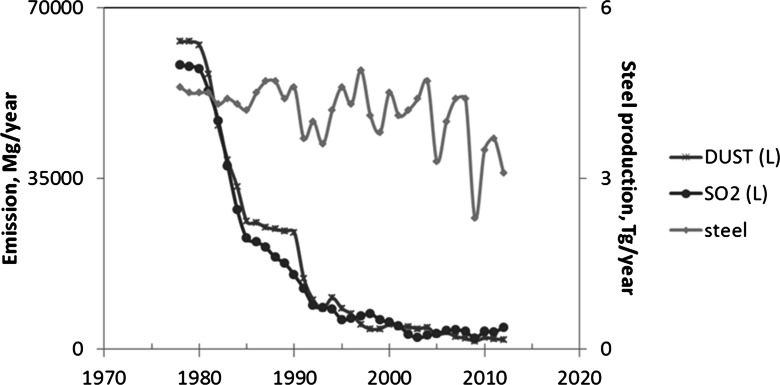
Instrumental records of sulfur dioxide and dust emission and steel production by “Huta Katowice” steelworks

The sampling sites are located in proximity to Huta Katowice steelworks (current name, ArcelorMittal Poland) in Dąbrowa Górnicza (50° 20′ 31″ N 19° 16′ 1″ E; Fig. 1), which opened in 1975. This is one of the largest steelworks in the southern part of Poland. The highest levels of pollutant emissions from this plant were recorded in 1978–1980 (Fig. 2), while a significant decrease in dust and sulfur dioxide emissions was noted from 1981 to 1984. These changes were the effect of correcting the design errors and implementing measures to improve the effectiveness of the protective devices. The most reduction of industrial pollution emission in Huta Katowice is connected with the modernization of the industrial sector and also with EU legislation and the application of restrictive governmental regulations on emissions. Since 1978, the most significant reduction of industrial dust emission was noted from 1980 to 1985 and from 1990 to 1992, whereas the most significant reduction of sulfur dioxide emission was noted from 1980 to 1995, respectively. The tendency in the emission of pollution is to decrease from year to year. The area surrounding Huta Katowice has been used as a research testing ground for many years (e.g., Rostański 1990; Wyżgolik and Michalski 1993; Pomierny and Ciepał 2004; Sensuła et al. 2015a, b), but there have never been any stable isotope analysis performed on tree rings.

The study area was dominated by winds from the southwest and the west that is why a comparative site in Olesno (OL; Fig. 1) was selected in the area located around 100 km NW from the Silesian industrial emitters. However, the impact of pollution from long-range transport route could not be fully excluded. The choice of location allowed the homogeneity of the species, age, climatic conditions, and habitat to minimize the impact of pollution from the industrialized part of the Upper Silesian region.

### Meteorological and Pollution Data

**Fig. 3:**
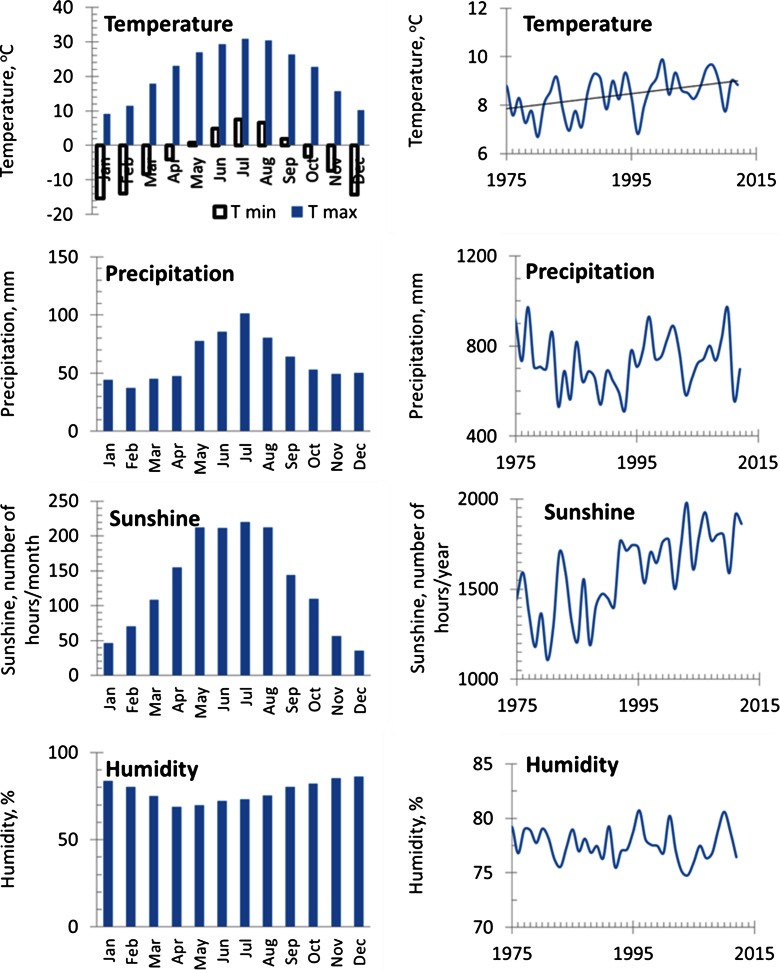
Climatic conditions in the investigated area from 1975 to 2012, mean monthly maximum and minimum temperature, annual mean temperature, mean monthly and annual sum of precipitation, mean monthly and annual sunshine, and monthly and annual mean of relative humidity

The period from 1975 to 2012 AD was characterized in the regional climate records (the meteorological station in Katowice) by an annual mean temperature about 8 °C (data range from 6.6 to 9.8 °C/year), and a mean annual sum of precipitation of around 743 mm (data range from 515 to 1.011 mm/year) mean annual number of sunshine hours is approximately 1580 (data range from 1107 to 1978) and relative humidity around 77 % (data range from 74 to 80 %). The lowest precipitation was observed between mid-1980s and mid-1990s (Fig. 3). The vegetative period begins in April and lasts until September. The meteorological data were obtained thanks to the Polish Institute of Meteorology and Water Management (IMGW-PIB).

Sulfur dioxide emission ranged from around 63,000 Mg/year in 1978 to 4500 Mg/year in 2012, whereas industrial dust emission ranged from 58,000 Mg/year in 1978 to ca. 2000 Mg/year in 2012. A significant correlation was detected between sulfur dioxide and industrial dust emission (*r* = 0.74, *n* = 35, *p* < 0.05). The production of steel ranged from approximately 4.6 Tg/year in 1978 to approximately 3 Tg/year in 2012 (Fig. 2). The data of pollution emitted by the steelworks (sulfur dioxide and dusts) were obtained from the archives kindly provided by Huta Katowice. For the statistical calculation, Statistica 10.0 software was applied.

### Sampling and Chemical Pre-Treatment

Scots pines (*P. sylvestris* L.) is the main tree species in both forests and thus was selected for this research. Dendrochronological research was the first part of BIOPOL project (Sensuła et al. 2015a, b; Sensuła 2015). It should be mentioned that the examined specimens were healthy, dominant in the stand, and had survived the adverse effects of industrial pollution. In the summer of 2012, cores of the trees were collected at the breast height using a 5-mm-diameter increment borer. In order to avoid a different dendroecological reaction of juvenile wood, an attempt was made to select pine stands aged between 90 and 100 years (felling age of Scots pine). The analyzed samples covered the time span from 1975 to 2012 AD. The α-cellulose samples were extracted from increment cores of ten representative trees. The absolutely dated annual tree rings were manually separated as thin silvers and pooled and homogenized. The α-cellulose samples were extracted by applying the procedures based on Green’s method (1963) used in the Mass Spectrometry Laboratory of Silesian University of Technology (Pazdur et al. 2007, 2013; Sensuła et al. 2011a, b; Sensuła and Pazdur 2013a, b). This method includes the following steps: the removal of lignin, the processing of the holocellulose to α-cellulose, bleaching, neutralizing, and drying. The hydrolysis of α-cellulose by 72 % H_2_SO_4_ was performed under the conditions described by Chambat et al. (1997) and was adopted by author in the Mass Spectrometry Laboratory of Silesian University of Technology in Poland (Sensuła et al. 2011b). To ensure homogeneity, the chemical pre-treatment was carried in an ultrasonic bath. Pre-tests made in the Mass Spectrometry Laboratory of Silesian University of Technology in Poland have shown that the precision of the carbon isotope measurements in α-cellulose was less than in glucose. The precision on triplicates was ±0.3‰ (*n* = 50, for cellulose) and ±0.15‰ (*n* = 50, for glucose) (Sensuła 2016). In these measurements, wood (C-5) and α-cellulose (C-3) reference materials from IAEA were used.

### Isotopic Measurements

In order to determine the δ^13^C values, the samples (0.060 mg) were loaded into zin capsules and combusted at a temperature of 1100 °C, and then, CO_2_ was separated in gas chromatography column in the elemental analyzer (EuroVector). In order to determine the δ^18^O values, the samples (0.095 mg) were loaded into silver capsules. To displace moisture-containing air in the glucose samples, the samples were heated over 24 h in a vacuum line (60 °C), and after that, they were put into a special air-filled homemade dry box before stable isotope ratio determination (Sensuła et al. 2011b). The samples were converted to CO by pyrolysis at a temperature of 1350 °C and separated in gas chromatography column in the elemental analyzer (EuroVector).

The stable oxygen and carbon isotope compositions of the samples were determined using an Isoprime continuous flow isotope ratio mass spectrometer (GV Instruments, Manchester, UK) at the Mass Spectrometry Laboratory of the Silesian University of Technology.

The relative deviation of the isotopic composition is expressed, in parts per thousand (‰), as

$$ \delta =\left({R}_{\mathrm{sample}}/{R}_{\mathrm{standard}}-1\right)\kern0.5em \times \kern0.5em 1000, $$

where *R*_sample_ and *R*_standard_ are the ratios of the heavy to the light isotope concentration in the sample and in the standard, respectively. The δ^13^C results are reported in values relative to VPDB, whereas the δ^18^O results are reported in values relative to VSMOW.

## Results and Discussion

**Fig. 4:**
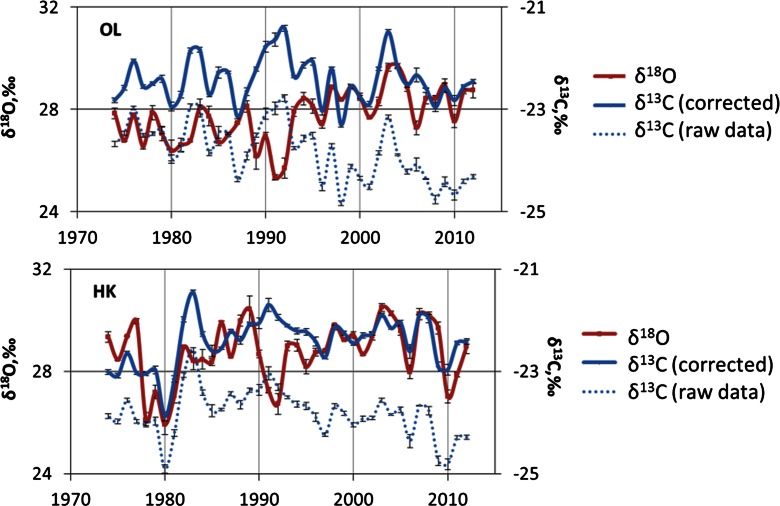
Spatial and temporal variations of δ^18^O and δ^13^C in pine growing in the forests located in proximity to the steelworks (HK) and the comparative site (OL)

Each of the pine tree ring isotopic chronologies constructed in this study is a local grown pattern of the pine population. These isotopic records are the result of the response of the trees to variations of various environmental factors, inter alia climatic drivers, and human activities. The pattern of spatial and time variabilities of δ^13^C and δ^13^O in pine growing in two forests (in proximity to the steelworks in Dabrowa Gornicza (HK) and in comparative site (OL)) are illustrated (Fig. 4) and summarized in Table 1 (*n* = 39).

**Table 1:** Comparison of stable carbon and oxygen isotope composition of glucose samples from pine growing in the forests located in proximity to the steelworks (HK) and the comparative site (OL)

Isotope	Site	Average	Standard deviation	Minimum	Maximum
δ^18^O, ‰	HK	28.8	1.2	25.9	30.5
δ^18^O, ‰	OL	27.7	1.1	25.3	29.7
δ^13^C, ‰	HK	−23.81	0.47	−24.9	−22.6
δ^13^C, ‰	OL	−23.81	0.56	−24.8	−22.8

### δ^13^C Chronologies

According to the NOAA, the average global atmospheric CO_2_ concentration has risen from 331 ppm in 1975 to 393 ppm in 2012. The observed anthropogenic impact on the carbon cycle that is mainly related to various global industrial activities (Marland et al. 2008) has caused changes in the isotopic composition of carbon in global air atmosphere (Martin et al. 1988; Ferrio et al. 2003; Pazdur et al. 2013). Elevated CO_2_ significantly decreases δ^13^C in the air. The δ^13^C present in air is about −8.3‰. Plants grown at the higher level of CO_2_ concentration had a more negative δ^13^C than plants grown at the lower concentration. In pine, similarly as in C_3_ plants, CO_2_ usually limits photosynthesis, and thus, an increase in CO_2_ results in greater photosynthetic rates.

Raw δ^13^C data were corrected to a pre-industrial atmospheric δ^13^C base value of 6.4‰ using the data from McCarroll and Loader (2004), because decreasing δ^13^C in the air and the biosphere is associated with the increasing of CO_2_ in the atmosphere (Craig 1954; Farquhar and Lloyd 1993; Saurer et al. 2002; McCarroll and Loader 2004; McCarroll et al. 2009; Pazdur et al. 2007, 2013; Keeling et al. 2010; Rinne et al. 2010; Sensuła and Pazdur 2013a, b; Battipaglia et al. 2013). This correction lifts the δ^13^C values (Fig. 4).

A depletion of δ^13^C values may be also due to fossil fuel emissions connected with local sources of pollution (inter alia industrial factories, vehicles, and law stack emission of CO_2_). The investigated site HK is located in vicinity of industrial company, whereas comparative site OL is located near Olesno, about 100 km NW from the heavily industrialized area of Silesia.

According to the Regional Inspectorate for Environmental Protection, the air quality has been deteriorating in Olesno from year to year, especially with the beginning of the heating season. In this region, similarly like in the most cities in Poland, depletion in δ^13^C may be connected with low stack emissions of CO_2_ from homes and housing energy because many people in Olesno did not only burn coal in furnaces but also plastic bottles and clothing in their homes. As a result, the air contains the toxic components, including toxic dioxins, furans, heavy metals, and benzene, which influence human, animal, and plant lives. The trends of δ^13^C in pine growing in both forests coincide (*r* = 0.42, *p* < 0.05). The gradient changes of δ^13^C can be observed from year to year.

### δ^18^O Chronologies

δ^18^O values in glucose samples from pine growing in the forests located in proximity to the steelworks (HK) show also a correlation between the δ^18^O values in glucose samples from pine growing in the comparative site (OLE; *r* = 0.53, *p* < 0.05). The gradient changes of δ^18^O from year to year can be observed. Spatial inhomogeneity of δ^18^O changes is a function of the type of source of pollution (Fig. 4). In general, the higher values of δ^18^O are observed in proximity to the steelworks than in the comparative site. Different trends between these two chronologies are observed for the period of time from 1975 and 1990. From 1975 to the 1990s, significant and sharp decreases of sulfur dioxide and dust emission by Huta Katowice steelworks have been noted (Fig. 2). A similar variability of δ^18^O trends, as tree response to environmental changes, has been observed since 1990 AD. Since the 1990s, the increase of sulfur dioxide and dust emission has been slighter in the vicinity of Huta Katowice. This confirms that industrial pollution may have an impact on oxygen isotope fractionation in pine and the presence of the non-climatic signal in the δ^18^O chronology of HK.

### Combined Measurement of Carbon and Oxygen Isotope Ratio

Combined measurement of carbon and oxygen isotope ratio gives information about the changes in stomata conductance (*g*_*s*_) or/and changes in photosynthetic capacity (*A*_max_—the average maximum net photosynthesis at ambient CO_2_ concentration under optimal environmental conditions). In other words, the model (Scheidegger et al. 2000; Farquhar and Lloyd 1993) indicates which factor responded more strongly. Stomatal regulation of water loss concerns plant response to variation in the growth environment. These changes can be also described by carbon discrimination (Δ^13^C), which is a photosynthesis-weighted integrator of carbon supply and demand (Farquhar and Lloyd 1993). The isotopic discrimination in photosynthesis can be described as the model of (Farquhar and Lloyd 1993)

$$ \begin{array}{l}\Delta {}^{13}\mathrm{C}=\frac{\updelta^{13}{\mathrm{C}}_{\mathrm{air}}-{\updelta}^{13}{\mathrm{C}}_{\mathrm{plant}}}{1+\frac{\updelta^{13}{\mathrm{C}}_{\mathrm{plant}}}{1000}}\hfill \\ {}{\Delta}^{13}\mathrm{C}\kern0.5em =\kern0.5em a\kern0.5em +\left(b-a\right){C}_i/{C}_a\hfill \end{array} $$

where *C*_*i*_ is the intercellular CO_2_ concentration, *C*_*a*_ is the ambient CO_2_ concentration, *a* (ca. 4.4‰) is the discrimination against ^13^CO_2_ during CO_2_ diffusion through stomata, and *b* (ca. 27‰) is the discrimination associated with carboxylation.

Positive correlation between δ^13^C and δ^18^O is predicted when Δ^13^C is driven by reduction in stomata conductance and not much changes in *A*_max_, whereas the upward trend reflects an increase in *A*_max_ with no or minor reaction in *g*_*s*_. No relationship between δ^13^C and δ^18^O is expected when variation in Δ^13^C is driven by changes in photosynthetic capacity alone, because δ^18^O is not affected by photosynthetic capacity. If variation in Δ^13^C is driven by increases in both stomata conductance and photosynthetic capacity, then the change in δ^18^O per unit change in Δ^13^C will be greater than if stomata conductance alone had increased (Barbour et al. 2000, 2002). These analyses are only qualitative. The analysis of combined measurement of carbon an oxygen isotope ratio (Fig. 4) shows positive correlation between δ^13^C and δ^18^O in two periods of time (before mid-1980s and after mid-1990s). According to Saurer and Siegwolf (2007), the stronger response of *A*_max_ indicates that some species were able to enhance biomass accumulation due to increasing CO_2_ during twentieth century, whereas other plants responded more strongly with reduced stomatal conductance and less transpiration and water loss.

### Climatic Data and Isotope Data

**Fig. 5:**
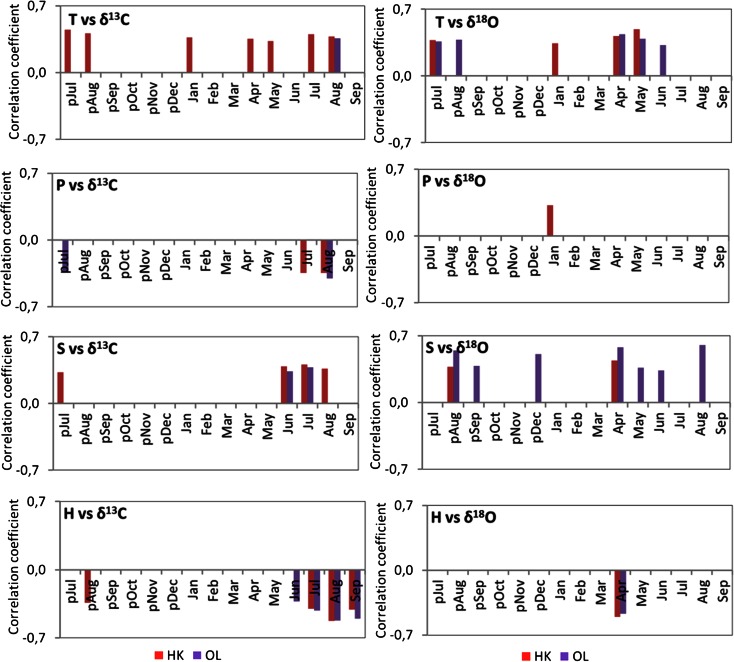
The significant correlation coefficients (at 95 % confidence level) between the monthly mean precipitation (P), monthly sunshine (S), mean temperature (T), monthly humidity (H) from previous July to the current September, and stable C and O isotope compositions of pine growing in the vicinity of the steelworks (HK) and the comparative site (OL) from 1975 to 2012 AD)

The resulting isotopic chronologies were correlated against meteorological parameters from the nearest meteorological station in Katowice (Fig. 5). Relations between climate and the isotopic composition exhibit spatiotemporal diversity. The presented analyses indicate that the months for each parameter are those that give the highest correlation (at the 95 % significance level) with the isotope data (Fig. 5). Composite correlation diagrams demonstrate the relative dominance of monthly climate parameters (mean temperature, sum of precipitation, sunshine, and relative humidity) on isotope fractionation and show the sensitivity of trees to the climate of specific months (since July of the previous year till September of the given year). The sensitivity of trees of both populations is different. The impact of weather conditions of the previous year on the growth of the current year is observed.

### Climate and δ^13^C Chronologies

Strong, highly significant statistical relationship carbon isotopes with summer climate variables were identified (Fig. 5). Temperature and sunshine, especially in late summer and early autumn, have a positive impact on δ^13^C. Positive relationships between mean temperature in summer of previous and current years and temperature in January and May and carbon isotope composition are observed only in HK population, whereas only a single positive relationship between late summer temperature and carbon isotope composition is observed in OL population. Positive relationships between summer sunshine and carbon isotope composition are similar at the both sampling sites. Also, negative relationships between autumn precipitation, summer and early autumn humidity, and carbon isotope composition are similar at the both sampling sites.

For carbon isotopes, fractionation is a function of the ratio of internal to external concentrations of CO_2_ and is thus a measure of the balance between stomatal conductance and photosynthetic rate.

The climatic controls are thus those factors that control stomatal conductance which are dominated by air relative humidity and soil moisture stratus and those that control photosynthetic rate which are dominated by light levels and leaf temperature (McCarroll and Loader 2004). A number of studies have linked the carbon isotopes in plants with relative humidity, rainfall, temperature, and sunshine (Stewart et al. 1995). Where stomatal conductance dominates, the controls include air relative humidity and soil moisture status, which is linked to antecedent precipitation. Where photosynthetic rate dominates, the dominant controls are irradiance and temperature. In areas where the dominant stress factor influencing trees is a combination of high temperature and low precipitation, then δ^13^C provides a strong indicator of the severity of those conditions. Under less extreme conditions, and where neither stomatal conductance nor photosynthetic rate is strongly dominant, the δ^13^C values will be influenced by all of these factors (Gagen et al. 2004; Zimmerman and Ehleringer 1990; McCarroll and Loader 2004). Lipp et al. (1991) analyzed that the relationships between the carbon stable isotope contents in dendrochronologically dated tree rings of firs and air temperature, relative humidity, and precipitation rate were investigated from 1959 to 1980. They found the δ^13^C values show significant correlations with temperature, humidity, and precipitation. Boettger et al (2014) analyzed the climate sensitivity of carbon isotope signatures in tree ring cellulose of *Abies alba* Mill. from a marginally industrialized area of Franconia (Germany) and reported a clear reduction in climate sensitivity of δ^13^C.

### Climate and δ^18^O Chronologies

Temperature and sunshine, especially temperature in autumn in the previous year and temperature in the first half of the current year, and solar radiation in autumn of previous year and spring of current year have a positive influence on δ^18^O, whereas humidity in April has a negative influence on δ^18^O. It has been found a single positive correlation between δ^18^O and temperature and precipitation in January in HK (Fig. 5).

Oxygen isotopes in tree rings reflect the isotopic composition of the source water and a number of bio-physiological factors, including bio-synthesis, xylem water-sucrose exchange, and leaf water evaporative enrichment (McCarroll and Loader 2004). Variable exchange with xylem (source) water during wood synthesis determines the relative strength of the source water and leaf enrichment signals.

The bio-physiological effects tend to be similar for a given species grown in the same environment (Anderson et al. 2002). Stable oxygen isotopic ratios record source water, which contains a temperature signal, and leaf transpiration, controlled dominantly by vapor pressure deficit. There are some direct temperature effects on evaporative enrichment including a small effect on the difference in vapor pressure of the water containing heavy and lighter isotopes; however, the dominant control on degree of leaf enrichment for δ^18^O is leaf to air vapor pressure deficit, which is controlled by air humidity rather than directly by temperature (Buhay et al. 1996). Robertson et al. (2001) found strong correlations between δ^18^O ratios from oak tree rings with both summer air relative humidity and the δ^18^O of precipitation, dominated by winter rainfall. Anderson et al. (2002) have used δ^18^O from fir trees in central Switzerland to reconstruct past variation in the δ^18^O of precipitation. Several studies report that the oxygen isotope ratio in tree ring reflects the oxygen isotope ratio of precipitation and can be sensitive to air temperature (Burk and Stuiver 1981), relative humidity (Edwards and Fritz 1986), transpiration rates (Barbour et al. 2000), and water balance (White et al. 1994), presumably all through contributions of source water (the xylem water in suberized stems) and leaf water enrichment. Few studies have linked the oxygen isotopic composition of tree ring cellulose with moisture or humidity. Saurer et al. (2002) found that the δ^18^O in tree rings was positively correlated with precipitation amount. Edwards and Fritz (1986) have concluded that the oxygen isotopic composition of cellulose recorded interannual variations in atmospheric humidity, whereas DeNiro and Cooper (1989) have reported no evidence of a humidity signal in δ^18^O in plants. Boettger et al (2014) analyzed that the climate sensitivity of oxygen isotope signatures in tree ring cellulose of *A. alba* Mill. reported that δ^18^O climate relations remain well pronounced after 1950.

### Industrial Dust and Sulfur Dioxide Emission Impacts on δ^18^O and δ^13^C

Besides different climatic factors, the response of trees to other environmental condition should be considered. During the last century, numerous human activities have been observed in different parts of ecosystem. These include increased not only atmospheric CO_2_ but also other atmospheric pollution, as well as changes in forest management. Analysis of climate data and δ^18^O and δ^13^C chronologies of pines growing in two forests suggests a presence of the non-climatic signal recorded in isotope composition of trees. The presence of the non-climatic signal in the isotopic chronology may be reflected in the correlation analysis par differences of *r* values between chronologies and climate parameters.

**Fig. 6:**
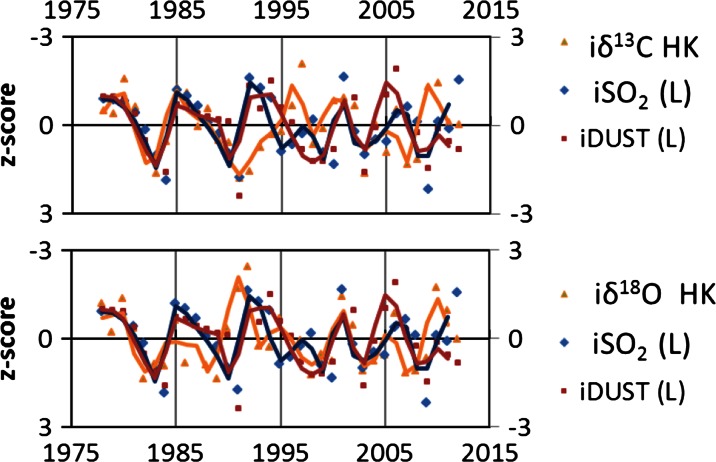
The impact of the sulfur dioxide and dust emission (*left*, *x* axis) on isotopic composition (*right*, *x* axis) of pine growing in the vicinity of steelworks (HK) (smoothed by 2-year average moving)

There is a lack of linear correlation between δ^18^O and δ^13^C and dust and sulfur dioxide emissions from steelwork. δ^18^O and δ^13^C values in glucose samples from the HK population and sulfur dioxide and industrial dust emission data have been normalized to *z*-scores to aid comparison (Fig. 6). Similar methods were used by Rinne et al. (2010). Due to large difference of amplitude of dust and sulfur dioxide emissions from 1978 to 2012 AD, all the values have been normalized for the following periods of time: (1) 1978–1983, (2) 1984–1990, (3) 1991–1994, (4) 1995–2004, and (5) 2005–2012.

The obtained normalized to *z*-scores showed a significant correlation between sulfur dioxide and industrial dust emission and normalized to *z*-scores maximum temperature in September (*r* = −0.44). Significant correlation was also noted between sulfur dioxide emission and normalized to *z*-scores precipitation in April (*r* = 0.43) and maximum temperature in January (−0.41).

The anticoincidence trends of δ^18^O and δ^13^C and dust and sulfur dioxide confirm that the decreases of dust and sulfur dioxide increase δ^18^O and δ^13^C. The illustrated results are smoothed by 2-year average moving, due to the initial wood development, and subsequently, the associated isotope fractionation values may be influenced by the environmental conditions of the previous year.

The results show that sulfur dioxide and also industrial dust emission may have an impact on the stable isotope composition. Comparing trends of sulfur dioxide, dust, and δ^18^O in glucose, it is observed that δ^18^O is in a better anticoincidence with industrial dust emission than with sulfur dioxide. In analyzing the trend of industrial dust emission and δ^13^C in glucose, a shift between values can be observed. This may mean that the sensitivity of trees is different and that trees may record a response to changes of dust emission over several years with certain delays. This phenomenon cannot be explained at this level of research.

According to the author’s knowledge, no analysis of the influence of sulfur dioxide and industrial dust emission on glucose hydrolysed from α-cellulose extracted from annual tree rings of pine growing in the forests located in proximity to the steelworks that cover the period of time of the last decades or any other post-industrialized or industrialized period was carried out. The literature reviews report that δ^13^C value in cellulose can be increased with the decrease of sulfur dioxide in the air. Sulfur dioxide emissions can increase the tree ring δ^13^C values by augmenting dark respiration and changing the photosynthate allocation and partitioning (Farquhar et al. 1989; Wagner and Wagner 2006; Rinne et al. 2010). The variation of δ^13^C and an overestimation of δ^13^C may be due to sulfur dioxide industrial emission in the area in proximity to the steelworks.

The literature background presents that the sulfur dioxide signal can be imprinted in the leaf water as a result of decreased loss of the lighter isotopes owing to decreased transpiration, but only approximately 42 % of the produced sucrose oxygen has a capacity for exchange with source water during cellulose synthesis (Dongmann et al. 1974; Hill et al. 1995; Roden et al. 2000; Roden 2008).

Boettger et al. (2014) report that SO_2_ background emissions of West Germany had influenced isotope signatures long before 1950. The relationships between isotope values and concentrations of SO_2_ and dust and other pollutants at the regional level during the period of 1979–2006 indicate that δ^13^C and δ^18^O were influenced primarily by SO_2_. Wagner and Wagner (2006) report that the long-term trend of δ^13^C showed an extraordinary increase in the period of 1945–1990 and a rapid decrease after 1990, whereas δ^18^O remained nearly constant. The increase of δ^13^C was explained by secondary fractionation caused by phytotoxicity of SO_2_. Two effects are mainly responsible for the secondary fractionation under SO_2_ exposure, increase of dark respiration and changes in photosynthate allocation and partitioning. Both effects did not influence δ^18^O. Rinne et al. (2010) concluded that the twentieth century δ^13^C and δ^18^O records of Woburn and the δ^13^C record of Windsor exhibit trends that we attribute to SO_2_ pollution. Since high ambient SO_2_ concentrations have been a rather common problem across Europe particularly before the mid-1980s, this pollutant may have had a stronger impact on the stable isotope ratios in tree rings than has been previously recognized. The combined physiological response to high pollution levels was less in δ^18^O than δ^13^C.

## Conclusions

The pollutant emission affects the condition and quality of human and plant lives. This study shows the first analysis of carbon and oxygen stable isotopes in glucose as the bio-indicators of CO_2_, sulfur dioxide, and industrial dust emission. Since the 1990s, the emission of pollutants was reduced in a majority of Polish and developing countries’ factories, whereas the level of energy production was similar to that prior to the 1990s. The conifers investigated in this study have grown for many years under the stress of industrial pollution. The variation of isotopic composition of Scots pine is the response of trees to various environmental factors (climate changes and anthropogenic activities). Relations between climate and industrial pollution emissions and isotopic composition of the analyzed populations exhibit spatiotemporal diversity, and the results of analysis for one stand should not be generalized to other sites. A comparison of δ^18^O and δ^13^C chronologies of the two pine populations (the first one growing in the vicinity of the steelworks and the second one growing far from the industrial emitters) suggests a presence of non-climatic signals recorded in the δ^18^O and δ^13^C values.

The variation of δ^13^C may be due to sulfur dioxide emission, especially in the population of pine growing in the vicinity of the steelworks. In effect, similar δ^13^C values in glucose in the sampling site in proximity of the steelworks and in the comparative site may be the result of the depletion of δ^13^C due to low stack emission and the road effect in the comparative site, and on the other hand, an overestimation of δ^13^C may be due to sulfur dioxide industrial emission in the area in proximity to the steelworks.

Comparing trends of sulfur dioxide, dust, and δ^18^O in glucose, it is observed that δ^13^C has anticoincidence with industrial dust emission and sulfur dioxide.

This approach can provide complementary information to reconstruct and analyze the environmental perturbations in the areas, where the reclamation of degraded landscapes takes place in the post-industrial period of time. The combination of several independent indicators constitutes a powerful tool as an example in environmental and ecological research.
